# Interrupted Reactions in the Synthesis of Subrubine Alkaloids

**DOI:** 10.1021/acscentsci.6c00767

**Published:** 2026-05-29

**Authors:** Jelena Golijanin, Logan R. Fagu, Andrei K. Yudin

**Affiliations:** Davenport Research Laboratories, Department of Chemistry, University of Toronto, Toronto, Ontario M5S 3H6, Canada

## Abstract

Efficient bio- and chemical total syntheses of subrubine alkaloids are achieved using interrupted construction of the diaza[3.3.3]propellane core.

## Graphical abstract

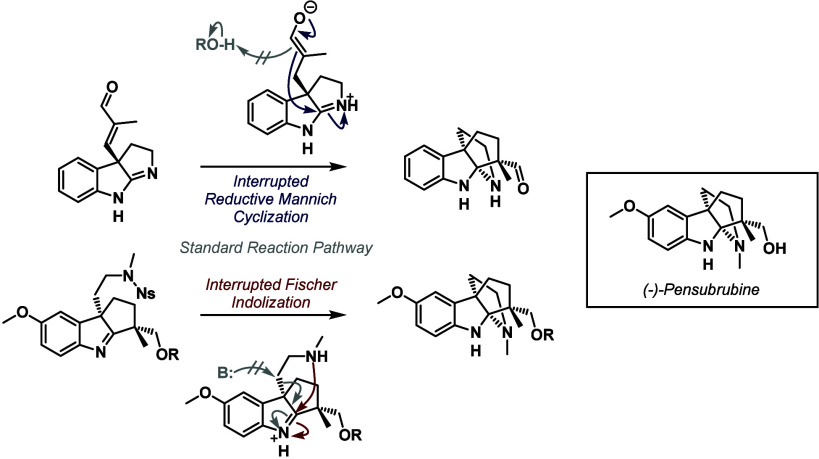

Interrupted reactions occur when a chemical process is diverted from its expected product to a new outcome. A common way to interrupt a reaction is to trap a transient intermediate.^1^ The discovery of an interrupted process is typically serendipitous, reflecting the difficulty in predicting the complex balance of relative rates. The utility of interrupted reactions is appreciated through their applications toward construction of novel scaffolds with potentially useful properties. This can be particularly enabling in the synthesis of complex natural products.^1^

The utility of interrupted reactions is appreciated through their applications toward construction of novel scaffolds with potentially useful properties. This can be particularly enabling in the synthesis of complex natural products.

In a recent issue of *ACS Central Science*, Garg, Tang, and co-workers utilize interrupted reactions in biosynthetic and chemical pathways to (−)-pensubrubine, an indole alkaloid in the subrubine family featuring a rare diaza[3.3.3]­propellane core.^2^ Indole alkaloids often possess potential anticancer, antimicrobial, and antiviral properties, making them attractive molecules for medicinal chemistry and chemical biology.^3^ Following the discovery of the first natural [3.3.3]­propellane modhephene in 1978, propellanes have gained attention as precursors for bicyclopentane (BCP) bioisosteres of benzene rings.^4,5^ Introduction of BCPs can lead to improved metabolic stability, aqueous solubility, and cellular membrane permeability.^5^ To date, there are numerous acid-catalyzed/thermal rearrangements, radical cyclizations, photochemical reactions, and metal-catalyzed processes that furnish [3.3.3]­propellanes.^6^ Comparatively less developed are strategies toward heteroatom-containing analogues. As a result, the number of biological studies surrounding these compounds generally remains scarce.

Inspired by previous work on interrupted reactions mediated by enzymes to access bioactive molecules, Garg, Tang, and co-workers began by searching for mutations in canonical yeast ene-reductases through a genome mining approach to identify localized groups of genes directly involved in producing certain metabolites (such as diaza[3.3.3]­propellanes in this case), called biosynthetic gene clusters (BGCs).^7^ Three types of BGCs were identified, with two corresponding to well-known transformations. The metabolite identities of the third, *sub* BGC, were probed further in several experiments, including gene deletion and isotope labeling. The results suggested that the SubF ene-reductase was responsible for interrupting an ene-reduction by rerouting an enolate intermediate toward a reductive Mannich cyclization, leading to several products in the newly discovered subrubine family, including (−)-pensubrubine (Figure 1). The biosynthesis established two important results. First, the work included the discovery of subrubines as microbial natural products, featuring the first report of a biosynthesized diaza[3.3.3]­propellane pyrrolidinoindoline core. The researchers found that the interrupted reaction step occurred most favorably with a tyrosine to phenylalanine residue mutation in the enzyme active site, consistent with previous observations that mutation aided in diversifying interrupted reactions promoted by ene-reductases.^7^ However, it was also found that mutation was not a necessary condition in achieving the desired reactivity, suggesting that the binding site of the enzyme could be further tailored toward alternative reactive pathways.

**Figure 1:**
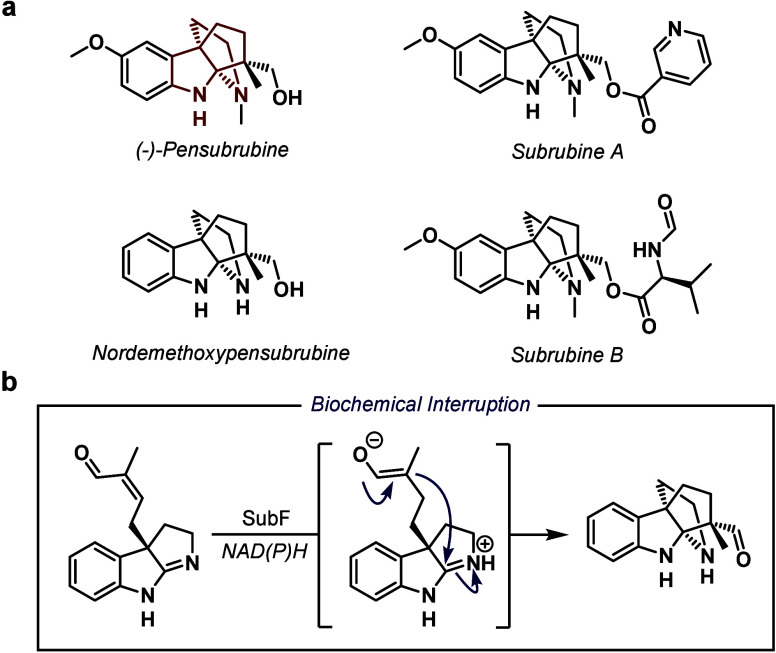
(a) Example molecules within the subrubine family, with the diaza[3.3.3]­propellane shown in red. (b) The interrupted enal reaction occurring in a biological setting.

Garg, Tang, and co-workers also performed an enantioselective total synthesis of (−)-pensubrubine to confirm the absolute stereochemistry of the microbial product. The authors utilized an interrupted Fischer indolization by introducing a quaternary center at C3. This makes rearomatization via deprotonation impossible and forces the indolenium ion to be captured by a nucleophile to furnish the diaza[3.3.3]­propellane in a stepwise manner (Figure 2).^8^ The total synthesis was short, involving only seven steps. The [3,3]-sigmatropic rearrangement to construct the core of the natural product was diastereoselective and high-yielding. The reported strategy is the first application of an interrupted Fischer indolization to achieve a diaza[3.3.3]­propellane core, drawing inspiration from biosynthesis to synthesize a motif difficult to access by other methods.

**Figure 2:**
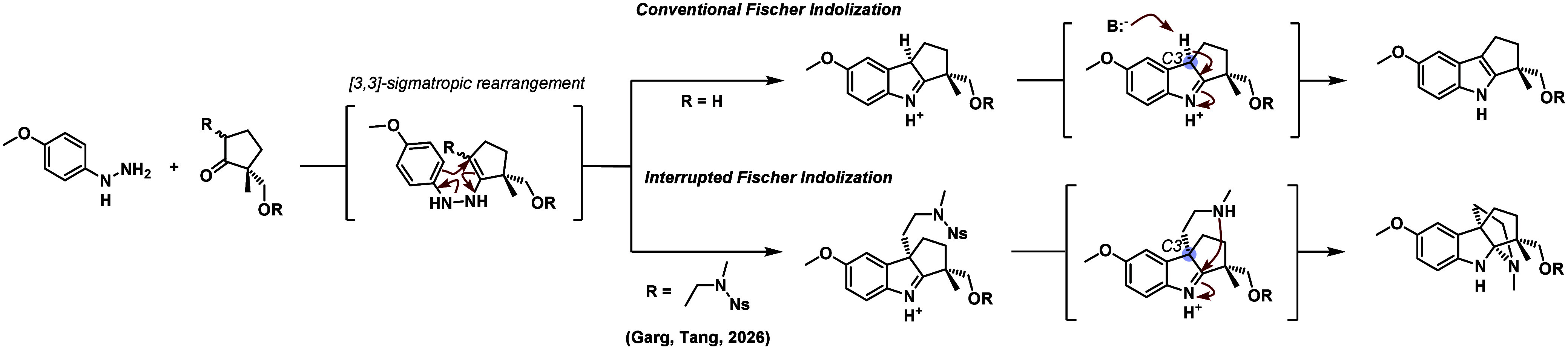
Key mechanistic aspects of the Fischer indole synthesis and the interrupted Fischer indolization pathways.

The reported strategy is the first application of an interrupted Fischer indolization to achieve a diaza[3.3.3]­propellane core, drawing inspiration from biosynthesis to synthesize a motif difficult to access by other methods.

Several conclusions can be drawn from the results of this work, with exciting implications for approaches toward diversified intermediates using interrupted reactions. In both the chemical and biosynthetic approaches, interrupted reactions were key to accessing the elusive diaza[3.3.3]­propellane core. This suggests interrupted reactions can be leveraged in other heteroatom-containing propellanes possessing other ring sizes, or motifs with different ring constituents. Notably, the rare propellane motif could be developed in additional classes of microbial natural products through enzyme diversification via directed biosynthesis. More broadly, similar predictive logic could be applied to complex systems to accelerate chemical discovery by expanding the number of available synthetic strategies and thus overcome current limitations in interrupted reaction planning or design.

In summary, Garg and co-workers have identified and synthesized the natural product (−)-pensubrubine via two pathways: (1) a biosynthesis involving an interrupted reductive Mannich cyclization; and (2) an enantioselective total synthesis involving a diastereoselective interrupted Fischer indolization. This study should encourage a broader search for biosynthesis-enabled interrupted reactions and will likely inform new synthetic strategies for scale-up applications and medicinal chemistry.
